# Reduced Post-ischemic Brain Injury in *Transient Receptor Potential Vanilloid 4* Knockout Mice

**DOI:** 10.3389/fnins.2020.00453

**Published:** 2020-05-12

**Authors:** Koji Tanaka, Shoji Matsumoto, Takeshi Yamada, Ryo Yamasaki, Makoto Suzuki, Mizuho A. Kido, Jun-Ichi Kira

**Affiliations:** ^1^Department of Neurology, Neurological Institute, Graduate School of Medical Sciences, Kyushu University, Fukuoka, Japan; ^2^Department of Comprehensive Strokology, School of Medicine, Fujita Health University, Toyoake, Japan; ^3^Department of Neurology, Saiseikai Fukuoka General Hospital, Fukuoka, Japan; ^4^Department of Pharmacology, Division of Molecular Pharmacology, Jichi Medical University, Shimotsuke, Japan; ^5^Department of Anatomy and Physiology, Faculty of Medicine, Saga University, Saga, Japan

**Keywords:** ischemic stroke, transient receptor potential vanilloid 4, knockout mice, brain edema, blood–brain barrier

## Abstract

**Background and Purpose:**

In the acute phase of ischemia-reperfusion, hypoperfusion associated with ischemia and reperfusion in microvascular regions and disruption of the blood–brain barrier (BBB) contribute to post-ischemic brain injury. We aimed to clarify whether brain injury following transient middle cerebral artery occlusion (tMCAO) is ameliorated in *Transient receptor potential vanilloid 4* knockout (*Trpv4^–/–^*) mice.

**Methods:**

tMCAO was induced in wild-type (WT) and *Trpv4^–/–^* mice aged 8–10 weeks. Ischemia-induced lesion volume was evaluated by 2,3,5-triphenyltetrazolium chloride staining at 24 h post-tMCAO. Tissue water content and Evans blue leakage in the ipsilateral hemisphere and a neurological score were evaluated at 48 h post-tMCAO. Transmission electron microscopy (TEM) was performed to assess the morphological changes in microvasculature in the ischemic lesions at 6 h post-tMCAO.

**Results:**

Compared with WT mice, *Trpv4^–/–^* mice showed reduced ischemia-induced lesion volume and reduced water content and Evans blue leakage in the ipsilateral hemisphere alongside milder neurological symptoms. The loss of zonula occludens-1 and occludin proteins in the ipsilateral hemisphere was attenuated in *Trpv4^–/–^* mice. TEM revealed that parenchymal microvessels in the ischemic lesion were compressed and narrowed by the swollen endfeet of astrocytes in WT mice, but these effects were markedly ameliorated in *Trpv4^–/–^* mice.

**Conclusion:**

The present results demonstrate that TRPV4 contributes to post-ischemic brain injury. The preserved microcirculation and BBB function shortly after reperfusion are the key neuroprotective roles of TRPV4 inhibition, which represents a promising target for the treatment of acute ischemic stroke.

## Introduction

Acute ischemic stroke secondary to large artery occlusion is a common and devastating condition that results in death or long-term disability in a high proportion of patients. Recent advances in revascularization therapies, including mechanical thrombectomy, have enabled rapid and effective recanalization for large artery occlusions in patients with acute ischemic stroke (Goyal et al., 2016). However, despite the high rates of early recanalization, some patients still experience poor outcomes. In these cases, lack of reperfusion due to blockade of the microcirculation leading to hypoperfusion associated with ischemia and reperfusion and disruption of the blood–brain barrier (BBB) may contribute to post-ischemic brain injury (Molina and Alvarez-Sabín, 2009).

Transient receptor potential vanilloid 4 (TRPV4), a member of the TRP vanilloid subfamily, is a non-selective cation channel permeable to Ca^2+^, Na^+^, and Mg^2+^ ions (Ramsey et al., 2006). TRPV4 was first described as a cellular osmotic sensor that detects extracellular hypo-osmolarity, and was subsequently shown to be activated by multiple stimuli. TRPV4 is widely expressed in the central nervous system (Nilius and Voets, 2013), and its localization on astrocyte endfeet plays an essential role in the regulation of vascular tone in brain microvessels (Dunn et al., 2013; Filosa et al., 2013). Enrichment of TRPV4 and aquaporins at the endfeet of astrocytes ensures that even small changes in conditions, such as extracellular ion concentrations, result in astroglial swelling, volume regulation, and reorganization of downstream signaling pathways (Ryskamp et al., 2015; Iuso and Križaj, 2016). Pharmacological blockade of TRPV4 results in reduction of brain ischemia-induced lesion volume through inhibition of matrix metalloprotease (MMP) activation, thereby providing protection against BBB disruption following transient focal cerebral ischemia (Li et al., 2013; Jie et al., 2015a, b, 2016). Moreover, TRPV4 contributes to Ca^2+^ influx in both astrocytes and neurons and to extracellular glutamate accumulation during peri-infarct depolarizations (Rakers et al., 2017). These findings indicate that TRPV4 has a large influence on ischemia-induced brain injury and indicate that inhibition of TRPV4 has a neuroprotective role in acute ischemic stroke. However, a recent report demonstrated exacerbation of ischemia-induced brain edema formation in *Trpv4* knockout (*Trpv4^–/–^*) mice (Pivonkova et al., 2018). Specifically, *Trpv4^–/–^* mice showed larger T2-hyperintensity lesions following transcranial permanent middle cerebral artery occlusion (pMCAO) compared with wild-type (WT) mice. Moreover, the report described a minimal involvement of TRPV4 in astrocyte volume regulation *in vivo*. Thus, the mechanism for the neuroprotective effects of TRPV4 in acute ischemic stroke remains inconclusive. Given this context, we aimed to investigate the differences in post-ischemic brain injury between *Trpv4^–/–^* and WT mice, and to clarify the mechanism for any identified differences using a transient MCAO (tMCAO) model in the C57BL/6N mouse strain.

## Materials and Methods

### Ethics Statement

The animal experiments were conducted in accordance with the National Institutes of Health Guide for the Care and Use of Laboratory Animals, and all procedures were approved by the Animal Ethics Committee of Kyushu University (Approval Nos. A28-08-4, A30-138-2, 26-60, and 1-48).

### Animals

*Trpv4^–/–^* mice were transferred from Jichi Medical University (Suzuki et al., 2003). C57BL/6N mice were purchased from Kyudo (Fukuoka, Japan). The *Trpv4^–/–^* mouse strain had been backcrossed with the C57BL/6N strain for >10 generations. Inbred adult male mice at 8–10 weeks of age (median [interquartile range] weight: 23.3 [21.7–24.9] g for WT mice and 24.1 [20.8–26.2] g for *Trpv4^–/–^* mice) were maintained in a temperature-controlled (22–25°C) specific pathogen-free animal facility on a 12-h/12-h light/dark cycle with free access to food and water.

### tMCAO Surgery

tMCAO was performed using a previously described method with modifications (Engel et al., 2011). Mice were anesthetized with 1.5–2.0% isoflurane (Escain; Mylan Seiyaku, Tokyo, Japan) in 30% oxygen-mixed gas under spontaneous respiration. Focal cerebral ischemia was induced by a silicone-coated 6-0 monofilament (#602356; Doccol, Redlands, CA, United States) inserted through a small incision in the right common carotid artery and then advanced through the internal carotid artery to occlude the origin of the MCA. The filament was withdrawn to allow reperfusion of the ischemic hemisphere. Regional cerebral blood flow in the right MCA territory was monitored by laser Doppler flowmetry (Omegaflo FLO-C1; Omegawave, Tokyo, Japan) to confirm vessel occlusion, shown by a rapid and dramatic decrease in brain perfusion (mean reduction: 70–80%) that remained stable until reperfusion. Body temperature was maintained at 37°C based on the rectal temperature. In sham-operated animals, the same procedure was performed except for insertion of the intraluminal filament. The optimal time of tMCAO was determined by a preliminarily experiment in which tMCAO was induced in WT mice for 15, 30, or 45 min. The lesion size at 24 h post-tMCAO became larger and more stable while the mortality rate increased with longer tMCAO induction (≈0% mortality for 15 min, ≈10% for 30 min, ≈20% for 45 min tMCAO). Based on the lesion size and mortality rate, 30 min tMCAO was used in this study.

### 2,3,5-Triphenyltetrazolium Chloride (TTC) Staining

Transient focal cerebral ischemia was induced in 12 *Trpv4^–/–^* and 12 WT mice. At 24 h post-tMCAO, mice were transcardially perfused with cold saline. The brain was removed immediately and cut into serial 1-mm-thick coronal sections. The sections were incubated in a 2% solution of TTC (Sigma-Aldrich Japan, Tokyo, Japan) for 15 min at 37°C. Images were captured with a Nikon D7100 digital camera (Nikon, Tokyo, Japan). Unstained areas were measured in TTC-stained sections using ImageJ software, version 1.46r (National Institutes of Health, Bethesda, MD, United States) to calculate the infarct volume.

### Tissue Water Content

Transient focal cerebral ischemia was induced in 12 *Trpv4^–/–^* and 12 WT mice. At 48 h post-tMCAO, mice were decapitated under anesthesia and the olfactory bulb, cerebellum, and brainstem were removed from each dissected brain. The ipsilateral and contralateral hemispheres were separated and their wet weights immediately recorded. The dry weights were determined after drying the tissues to a constant weight at 100°C for 48 h. Tissue water content was calculated as % H_2_O = (1 − dry weight/wet weight) × 100%.

### Evans Blue (EB) Extravasation

Transient focal cerebral ischemia was induced in 12 *Trpv4^–/–^* and 12 WT mice. At 46 h post-tMCAO, 2% EB dye (Sigma-Aldrich Japan) in normal saline was injected intraperitoneally at a dose of 4 ml/kg body weight. Subsequently, the mice were perfused with cold saline at 48 h post-tMCAO. The ipsilateral hemisphere was collected and incubated with 500 μl formamide (Sigma-Aldrich Japan) at room temperature for 24 h to extract EB. The concentration of dye in the extract was calculated by reference to a standard curve of EB in formamide quantified at 620 nm by spectrofluorophotometry (PerkinElmer Ensight; PerkinElmer, Waltham, MA, United States).

### Neurological Scoring

Neurological scores were evaluated at 24 and 48 h post-tMCAO in the *Trpv4^–/–^* and WT mice subjected to evaluation of tissue water content and EB leakage (*n* = 24 per group) using a scoring system modified from a neurological scoring system for sensorimotor deficits (Garcia et al., 1995). The highest possible score of 18 indicated no neurological deficit and the lowest score of 3 indicated the most severe impairment.

### Western Blot Analysis

Transient focal cerebral ischemia was induced or a sham operation performed in 9 *Trpv4^–/–^* and 9 WT mice. At 48 h post-tMCAO, mice were transcardially perfused with cold saline and a 1-mm coronal slice of the ipsilateral hemisphere at 4 mm posterior to the olfactory bulb was collected. The brain slice was homogenized in RIPA buffer (50 mM Tris-HCl pH 7.4, 150 mM NaCl, 5 mM EDTA, 1% Nonidet P-40, 1% sodium deoxycholate, 0.1% SDS, 1% aprotinin, 50 mM NaF, 0.1 mM Na_3_VO_4_, 1% Triton-X100) containing anti-phosphatase PhoSTOP (Roche, Mannheim, Germany) and then centrifuged at 10,000 × *g* for 10 min at 4°C. The protein sample was separated by 4–15% SDS-PAGE and transferred to a polyvinylidene fluoride membrane. The membrane was incubated with anti-zonula occludens (ZO)-1 (61-7300; Zymed Laboratories, San Francisco, CA, United States; 1:1,000 dilution), anti-occludin (ab167161; Abcam, Cambridge, MA, United States; 1:2,000 dilution), or anti-β-actin (A5441; Sigma-Aldrich Japan; 1:20,000 dilution) primary antibodies at 4°C overnight. After washing in Tris-buffered saline with 0.5% Tween-20, the membrane was incubated with a horseradish peroxidase-labeled secondary antibody (Abcam). Antibody-bound protein bands were visualized by enhanced chemiluminescence (ECL prime; Amersham Biosciences, Piscataway, NJ, United States). The protein levels were quantitatively analyzed using a ChemiDoc Touch Imaging System (Bio-Rad, Hercules, CA, United States) and Image Lab software version 5.2.1 (Bio-Rad). The amount of occludin was defined as the sum of the amounts of its dimeric (120 kDa) and monomeric (64 kDa) forms (Yanai et al., 2017). The protein levels of ZO-1 and occludin were normalized by those of β-actin. One mouse without surgery was used as an external control.

### Reverse Transcription-Quantitative Polymerase Chain Reaction (RT-qPCR)

Five *Trpv4^–/–^* and five WT mice without surgery were evaluated for their baseline gene expression levels of *Trpv4*, *Aqp4*, and *Kcnj10*. The mice were transcardially perfused with cold saline and a 1-mm-thick coronal slice of the hemisphere at 4 mm posterior to the olfactory bulb was collected. Total RNA was extracted using an RNeasy Mini Kit (Qiagen, Valencia, CA, United States). The A260/A280 ratio was measured using a spectrophotometer (Nanodrop 2000; Thermo Fisher Scientific, Wilmington, DE, United States) to determine the purity and concentration of the RNA. Complementary DNA was synthesized from the total RNA using ReverTra Ace reverse transcriptase (Toyobo, Osaka, Japan). Subsequently, RT-qPCR analysis was performed in a 7500 Fast Real-Time PCR System (Applied Biosystems, Foster City, CA, United States) using TaqMan Fast Universal PCR master mixture and TaqMan probes (Applied Biosystems) for *Trpv4* (Hs01099348_m1), *Aqp4* (Hs00242342_m1), *Kcnj10* (Hs01922935_s1), and *Gapdh* (Sp03785097_s1) under standard reaction conditions recommended by the manufacturer. The mRNA levels were normalized by those of *Gapdh*.

### Transmission Electron Microscopy (TEM)

Transient focal cerebral ischemia was induced in one *Trpv4^–/–^* and one WT mouse. At 6 h post-tMCAO (Kurisu et al., 2016), mice were transcardially perfused with 4% paraformaldehyde with 3.4% sucrose. Brain slices including the right striatum were fixed with 2.5% glutaraldehyde in 0.1 M cacodylate buffer at 4°C overnight, post-fixed with 1% OsO_4_ in 0.1 M cacodylate buffer on ice for 2 h, dehydrated in a graded ethanol series and propylene oxide, and embedded in epoxy resin. Ultrathin sections were cut at 80-nm thickness with an EM UC7 ultramicrotome (Leica Microsystems, Wetzlar, Germany) and stained with 2% uranyl acetate followed by a mixture of lead nitrate-lead acetate-lead citrate. The sections were observed with a Tecnai 20 electron microscope (FEI Co., Eindhoven, Netherlands) operated at an accelerating voltage of 200 kV and micrographs were recorded at a magnification of 5,000× for at least 10 microvasculatures.

### Statistical Analysis

Statistical analyses were performed using JMP version 9.0 software (SAS Institute, Cary, NC, United States). Data are expressed as the median and interquartile range. Statistically significant differences were evaluated by the Wilcoxon rank-sum test or the Kruskal–Wallis test with *post hoc* comparison by Dunn’s multiple comparison test. Values of *p* < 0.05 were considered to indicate statistical significance.

## Results

### Brain Injury After tMCAO Is Reduced in *Trpv4^–/–^* Mice

**FIGURE 1:**
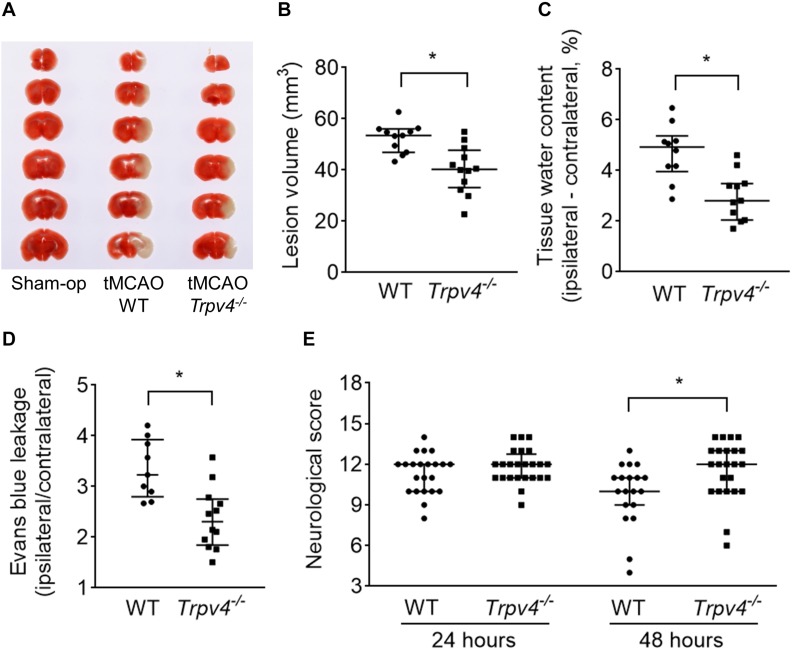
Effects of *Trpv4^–/–^* on neurological and pathophysiological findings. **(A,B)** Evaluation of ischemia-induced lesion volumes at 24 h post-tMCAO (one WT mouse died within 24 h; therefore, *n* = 11 for WT mice and *n* = 12 for *Trpv4^–/–^* mice). **(C)** Evaluation of tissue water content by subtraction of the contralateral hemisphere from the ipsilateral hemisphere at 48 h post-tMCAO (two WT mice and one *Trpv4^–/–^* mice died within 48 h; therefore, *n* = 10 for WT mice and *n* = 11 for *Trpv4^–/–^* mice). **(D)** Evans blue leakage presented as the ratio of leakage in the ipsilateral hemisphere to that in the contralateral hemisphere at 48 h post-tMCAO (three WT mice died within 48 h; therefore, *n* = 9 for WT mice and *n* = 12 for *Trpv4^–/–^* mice). **(E)** Evaluation of neurological scores at 24 and 48 h post-tMCAO (*n* = 22 for WT mice and *n* = 24 for *Trpv4^–/–^* mice at 24 h post-tMCAO; *n* = 19 for WT mice and *n* = 23 for *Trpv4^–/–^* mice at 48 h post-tMCAO). *p < 0.05.

To assess the ischemia-induced lesion volume, TTC staining was performed at 24 h post-tMCAO. The ischemia-induced lesion volume was significantly reduced in *Trpv4^–/–^* mice compared with WT mice (*p* = 0.002; Figures 1A,B). Because brain edema progressed to the maximum level on the second and third days, the tissue water content in the ipsilateral hemisphere was measured at 48 h post-tMCAO. The increase in tissue water in the ipsilateral hemisphere was significantly decreased in *Trpv4^–/–^* mice compared with WT mice (*p* = 0.004; Figure 1C). To assess the BBB function, EB leakage in the ipsilateral hemisphere was measured at 48 h post-tMCAO. The extent of EB leakage was significantly smaller in *Trpv4^–/–^* mice compared with WT mice (*p* = 0.002; Figure 1D). To assess the sensorimotor performance, the neurological score was evaluated at 24 and 48 h post-tMCAO in the two types of mice. Performance was impaired in both *Trpv4^–/–^* and WT mice after tMCAO with no significant difference between the groups at 24 h (12 [11–13] vs. 12 [10–12], *p* = 0.307; Figure 1E). However, *Trpv4^–/–^* mice showed significantly milder symptoms than WT mice at 48 h post-tMCAO (12 [10–13] vs. 10 [9–11], *p* = 0.009).

### Preservation of Tight Junction Proteins After tMCAO in *Trpv4^–/–^* Mice

**FIGURE 2:**
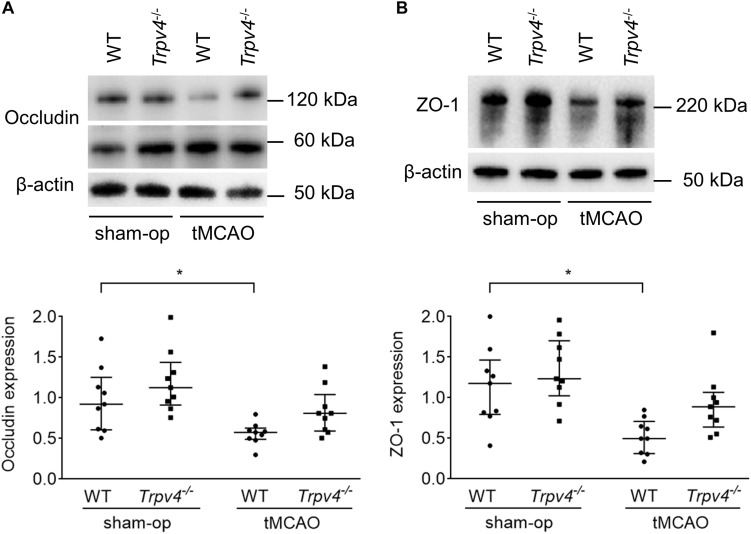
Effects of *Trpv4^–/–^* on changes in tight junction proteins. **(A,B)** Western blot analysis of occludin **(A)** and ZO-1 **(B)** protein levels in the ipsilateral hemisphere at 48 h after tMCAO or sham operation in WT and *Trpv4^–/–^* mice. Each group contained nine mice. Protein levels were corrected by β-actin. *p < 0.05.

To assess protein levels in tight junctions of the BBB, western blotting was performed at 48 h post-tMCAO (Figures 2A,B). The occludin and ZO-1 protein levels in the ipsilateral hemisphere were significantly lower in WT mice after tMCAO than after sham operation (*p* = 0.044 and *p* = 0.019, respectively), while these changes were not statistically significant in *Trpv4^–/–^* mice (*p* = 0.400 and *p* = 0.535, respectively).

### RT-qPCR Analysis of WT and *Trpv4^–/–^* Mouse Brains

**FIGURE 3:**
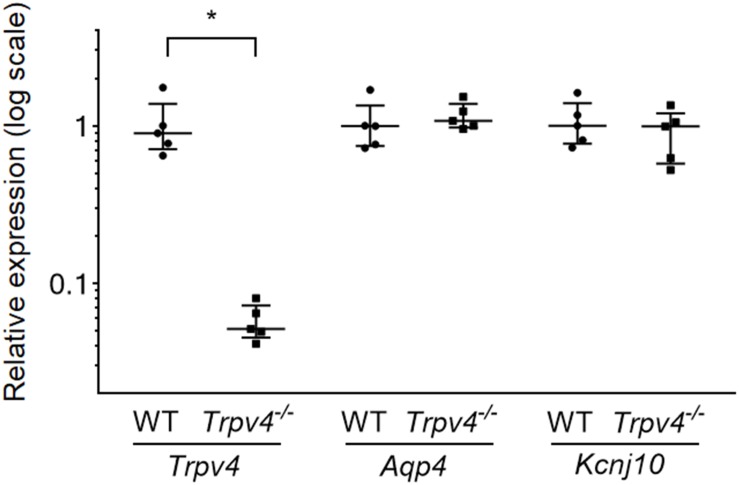
Expression levels of *Trpv4*, *Aqp4*, and *Kcnj10* in WT and *Trpv4^–/–^* mouse brains. RT-qPCR analyses were performed to measure the mRNA levels of *Trpv4*, *Aqp4*, and *Kcnj10* in the brains of WT and *Trpv4^–/–^* mice. Expression levels were corrected by *Gapdh*. Each group contained five mice. *p < 0.05.

The expression levels of *Trpv4* and genes associated with astrocyte volume regulation (*Aqp4* and *Kcnj10*) were evaluated by RT-qPCR. The data revealed significantly decreased expression of *Trpv4* in the *Trpv4^–/–^* mouse brain compared with the WT mouse brain (*p* = 0.009), while *Aqp4* and *Kcnj10* showed no differences (*p* = 0.347 and *p* = 0.465, respectively; Figure 3).

### Reduction in Astrocyte Endfeet Swelling in Microvessels After tMCAO in *Trpv4^–/–^* Mice

**FIGURE 4:**
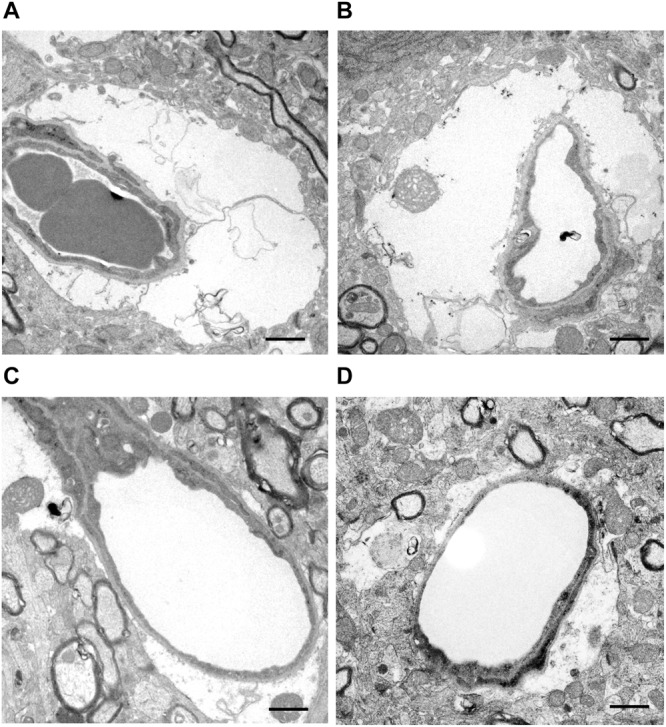
Representative images of microvascular morphological changes evaluated by TEM. **(A,B)** Microvessels are compressed and narrowed by swollen astrocyte endfeet in WT mice. **(C,D)** By contrast, the astrocyte endfeet swelling is milder and the residual vascular lumen is well-preserved in *Trpv4^–/–^* mice. One WT mouse and one *Trpv4^–/–^* mouse were assessed. Scale bars = 1 μm.

Given the highly polarized localization of TRPV4 in the astrocyte endfeet surrounding brain capillaries, microvascular morphological changes in the ischemic lesion were analyzed by TEM at 6 h post-tMCAO. As shown in Figure 4, there was evidence of widespread endfeet swelling of pericapillary astrocytes in most of the microvasculature in the ischemic lesion. The cytoplasm was expanded, and mitochondria in the endfeet were enlarged and degenerated with swollen cristae. The residual lumen of microvessels was narrowed through compression by the extensive swollen endfeet of perivascular astrocytes in WT mice, but this was markedly suppressed in *Trpv4^–/–^* mice.

## Discussion

This study demonstrated that *Trpv4^–/–^* mice showed reduced brain ischemia-induced lesion size and preserved BBB compared with WT mice in the acute phase. Astrocyte endfeet swelling and microvascular narrowing shortly after reperfusion were markedly suppressed in *Trpv4^–/–^* mice.

Previous research using ischemia-reperfusion animal models demonstrated decreased capillary blood flow with narrowed capillary diameters (Hauck et al., 2004). This microvessel narrowing was caused by swelling of perivascular astrocyte endfeet shortly after ischemia-reperfusion (Ito et al., 2011; Kurisu et al., 2016). Furthermore, the astrocyte endfeet swelling was markedly ameliorated by *Aqp4* knockout (Manley et al., 2000) or infusion of cold saline or mannitol (Ito et al., 2014; Kurisu et al., 2016), leading to reduced infarct volumes. Shimbo et al. (2017) demonstrated that infusion of hemoglobin-encapsulating liposomes, which were smaller than red blood cells and could pass through narrowed blood vessels, reduced the infarction size. These reports indicate that microvascular narrowing may contribute to hypoperfusion associated with ischemia and reperfusion. The mechanism for astrocyte endfeet swelling in acute ischemia was assumed to involve upregulated expression and polarized localization of AQP4 at astrocyte endfeet shortly after reperfusion (Steiner et al., 2012; Kurisu et al., 2016). The present study found similar mRNA expression patterns of *Aqp4* and *Kcnj10* between WT and *Trpv4^–/–^* mouse brains, in accordance with a previous study (Pivonkova et al., 2018). Blockade of TRPV4 altered hypothermia-induced surface localization of AQP4, despite there being no change in total protein expression levels (Salman et al., 2017). Thus, TRPV4 deficiency may reduce astrocyte endfeet swelling by inhibiting the AQP4 surge or AQP4 surface localization at astrocyte endfeet after ischemia-reperfusion.

This study demonstrated preserved BBB function shortly after reperfusion in *Trpv4^–/–^* mice, which was similar to that observed after pharmacological blockade of TRPV4 (Jie et al., 2015b). TRPV4-mediated Ca^2+^ influx led to activation of MMPs in lung tissue (Villalta et al., 2014), with subsequent disruption of tight junctions among alveolar epithelial cells (Dagenais et al., 2018). Therefore, TRPV4 deficiency may maintain the BBB via MMP inhibition to preserve tight junction proteins. A TRPV4 antagonist, GSK2798745, is currently being administered to patients in a phase II clinical trial for treatment of congestive heart failure-induced pulmonary edema (NTC02497937) (Stewart et al., 2020). EB leakage might have occurred because of BBB disruption and also because of ischemia-induced opening of connexin hemichannels in neurovascular unit constituent cells, making them permeable to EB dye (Orellana et al., 2013); however, TRPV4 has potential as a therapeutic target for post-ischemic brain injury from the perspective of BBB protection.

An important concern against clinical application of TRPV4 inhibition is that it may lead to increased lesion volumes (Pivonkova et al., 2018). However, this was inconsistent with the findings of the present study. The difference between the two studies may be because of the different model used (pMCAO vs. tMCAO). Although recanalization is considered the most important event, development of collateral circulation is another major determinant for severity of ischemic stroke with large artery occlusions. Dilation of leptomeningeal arteries occurs as early as 6 h after pMCAO via increased phosphorylation of endothelial nitric oxide synthase (eNOS) in response to shear stress (Iwasawa et al., 2018). Meanwhile, *Trpv4^–/–^* mice exhibited loss of shear stress-induced vasodilatation of the carotid artery (Hartmannsgruber et al., 2007), and endothelial downregulation of TRPV4 was shown to contribute to impairment of eNOS-mediated relaxation of the mesenteric artery (Boudaka et al., 2019). These findings suggest that TRPV4 deficiency may exacerbate ischemic brain injury through impairment of acute collateral development in cases of recanalization failure, such as the pMCAO model.

The neuroprotective effects of TRPV4 inhibition may not be solely derived from astrocytic TRPV4 as suggested in a previous report (Pivonkova et al., 2018), because TRPV4 is also expressed in endothelial cells and neutrophils. TRPV4 deficiency prevented neutrophil responses to proinflammatory stimuli, including formation of reactive oxygen species (ROS), adhesion, chemotaxis to break the alveolar–capillary barrier, and development of acute lung injury (Yin et al., 2016). Neutrophils play an important role in acute stroke pathology, wherein reperfusion provides neutrophils with access to the vulnerable brain (Stowe et al., 2009). TRPV4 deficiency or blockade may suppress neutrophil activation to break the BBB. Moreover, free radical damage was shown to play a key role in ischemia-reperfusion injury (Sun et al., 2018). Activation of TRPV4 enhanced oxidative stress in hippocampal neurons (Hong et al., 2016), and TRPV4 was simultaneously activated by ROS (Badr et al., 2016; Suresh et al., 2018). These observations indicate that TRPV4 and free radicals form a positive feedback loop that is involved in neurotoxicity. Thus, blockade of TRPV4 may have other neuroprotective effects in terms of ROS-mediated brain injury.

This study has several limitations. First, the mouse groups were not randomized for surgery and other tests because of the study design, which may have caused some selection biases. Second, in the TEM analysis, we could not perform a statistical evaluation because of a shortage in the number of detected microvessels in our data. Therefore, we qualitatively described the main observed findings in terms of microvascular morphological changes. Third, the neuroprotective effects in *Trpv4^–/–^* mice were only assessed in the acute phase of transient focal cerebral ischemia because most of the mice showed continuous body weight loss that exceeded 20% by 48 h post-tMCAO and thus met the humane endpoint for laboratory animals (Science Council of Japan, 2006). Therefore, the effects of *Tprv4* deficiency on chronic ischemia and delayed ischemic neurological deficits remain to be established. Activation of TRPV4 via 4α-phorbol 12,13-didecanoate was reported to contribute to functional recovery through angiogenesis and neurogenesis at 5 days post-tMCAO (Chen et al., 2018). Further investigations are needed to confirm whether long-term inhibition of TRPV4 in the subacute to convalescent phases plays a neuroprotective role. Finally, we only examined the expression of two genes that regulate astrocyte volume, *Aqp4* and *Kcnj10* (Iuso and Križaj, 2016); therefore, further unbiased gene expression studies using microarray methodology are necessary to reveal any gene expression changes that contribute to ischemia-resistance due to knockout of *Trpv4*.

## Conclusion

TRPV4 contributes to post-ischemic brain injury following transient focal cerebral ischemia. The preserved BBB function and microcirculation shortly after reperfusion are the key neuroprotective roles of TRPV4 inhibition, which represents a promising target for the treatment of acute ischemic stroke.

## Data Availability Statement

The datasets generated for this study are available on request to the corresponding author.

## Ethics Statement

The animal study was reviewed and approved by Animal Ethics Committee of Kyushu University.

## Author Contributions

KT, SM, and TY made substantial contributions to the concept and design of the study and analysis and interpretation of data. KT acquired data and drafted the manuscript. SM, TY, MS, MK, RY, and J-IK revised the manuscript critically for important intellectual content. KT approved the version to be published.

## Conflict of Interest

The authors declare that the research was conducted in the absence of any commercial or financial relationships that could be construed as a potential conflict of interest.
